# Supplementary material for: Red and Green Quantum Dot Color Filter for Full-Color Micro-LED Arrays
Source: Micromachines (Basel). 2022 Apr 10;13(4):595. doi: 10.3390/mi13040595 (PMC9029460; doi:10.3390/mi13040595)
Supplement: Supplementary file 1 [file micromachines-13-00595-s001.zip › micromachines-1614160-supplementary.pdf]

Article

Red and Green Quantum Dot Color Filter for Full-color Micro-LED Arrays

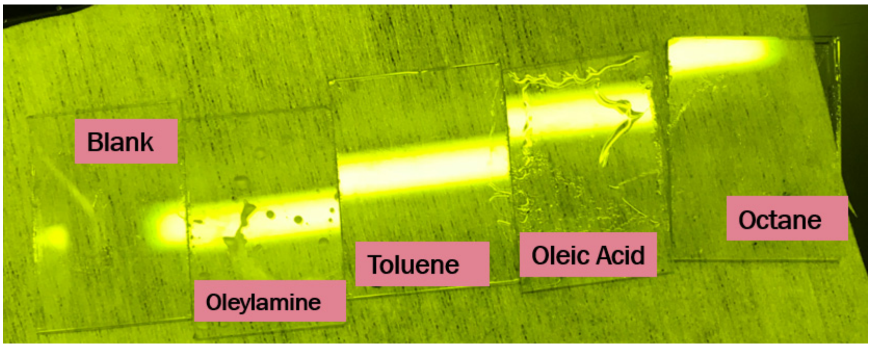

Figure S1. Chemical compatibility of different solvent for SU-8 photoresist.
